# The Role of Inflammation in Age-Related Macular Degeneration—Therapeutic Landscapes in Geographic Atrophy

**DOI:** 10.3390/cells12162092

**Published:** 2023-08-18

**Authors:** Grace A. Borchert, Hoda Shamsnajafabadi, Monica L. Hu, Samantha R. De Silva, Susan M. Downes, Robert E. MacLaren, Kanmin Xue, Jasmina Cehajic-Kapetanovic

**Affiliations:** 1 Nuffield Laboratory of Ophthalmology, Nuffield Department of Clinical Neurosciences, Oxford University, Oxford OX3 9DU, UK; 2 Oxford Eye Hospital, Oxford University NHS Foundation Trust, Oxford OX3 9DU, UK

**Keywords:** inflammation, AMD, age-related macular degeneration, geographic atrophy, gene therapy, optogenetics

## Abstract

Age-related macular degeneration (AMD) is the leading cause of vision loss and visual impairment in people over 50 years of age. In the current therapeutic landscape, intravitreal anti-vascular endothelial growth factor (anti-VEGF) therapies have been central to the management of neovascular AMD (also known as wet AMD), whereas treatments for geographic atrophy have lagged behind. Several therapeutic approaches are being developed for geographic atrophy with the goal of either slowing down disease progression or reversing sight loss. Such strategies target the inflammatory pathways, complement cascade, visual cycle or neuroprotective mechanisms to slow down the degeneration. In addition, retinal implants have been tried for vision restoration and stem cell therapies for potentially a dual purpose of slowing down the degeneration and restoring visual function. In particular, therapies focusing on the complement pathway have shown promising results with the FDA approved pegcetacoplan, a complement C3 inhibitor, and avacincaptad pegol, a complement C5 inhibitor. In this review, we discuss the mechanisms of inflammation in AMD and outline the therapeutic landscapes of atrophy AMD. Improved understanding of the various pathway components and their interplay in this complex neuroinflammatory degeneration will guide the development of current and future therapeutic options, such as optogenetic therapy.

## 1. Introduction

Age-related macular degeneration (AMD) is a neurodegenerative disease that affects the choroid, Bruch’s membrane, retinal pigment epithelium (RPE) and photoreceptor complex of the macula, the central cone-rich region of the retina, resulting in loss of central vision in the centre of the visual field and the ability to discern fine details. It is the leading cause of visual disability in people over the age of 50 years in the developed world and third globally [1]. Visual impairment associated with AMD has a significant impact on the quality of life, affecting an individual’s independence with high burden on healthcare systems [2,3]. The prevalence of AMD is increasing as the population ages [4]. This highlights its importance of AMD as a public health priority [5].

**Figure 1:**
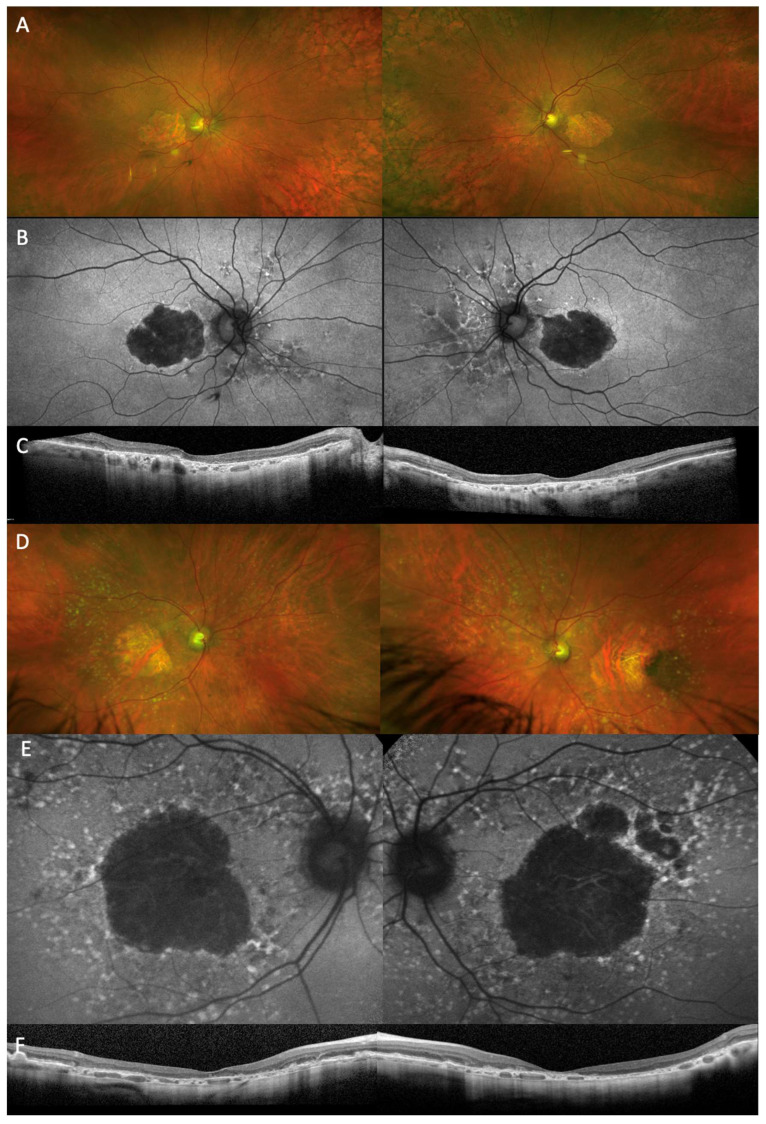
Multimodal imaging of geographic atrophy in a typical case of AMD (**A**–**C**) and in an AMD subtype with a confirmed genetic mutation in complement factor I (CFI) which is currently targeted in gene therapy trial. (**D**–**F**). Optos widefield (**A**,**D**) and fundus autofluorescence (**B**,**E**) images show well-demarcated areas of central retinal atrophy. There are drusenoid deposits at the macular and periphery typically seen with complement factor I deficiency phenotype (**D**,**E**). Macular optical coherence tomography (**C**,**F**) confirms central retinal atrophy predominantly of the outer retinal layers, the RPE and the choricocapillaris.

Early AMD is generally asymptomatic, but individuals may be affected by delayed in dark adaptation and increased photo stress recovery times [6,7]. With the onset of more significant retinal degeneration, the individual may experience blurred vision, distortion of straight lines, difficulty with reading or a central scotoma. Some patients notice changes in colour perception with difficulty distinguishing objects of similar colours or hue. In neovascular AMD develops, the patient may experience subacute onset of distortion or loss of central vision. AMD can be classified into early, intermediate and late stages, the latter being further divided into neovascular AMD and geographic atrophy [8,9]. Early AMD has clinical features of medium drusen >63 μm and <125 μm and no AMD pigmentary abnormalities. Intermediate AMD patients have large drusen (>125 μm) or pigmentary abnormalities associated with medium drusen (≥63 to <125 μm). Late AMD has neovascular AMD and/or any geographic atrophy. Multimodal imaging fundus photographs, fundus autofluorescence and spectral-domain optical coherence tomography (OCT) (Figure 1) permit further classification of AMD and are used to assess drusen subtypes, the presence of subretinal and intraretinal fluid, RPE changes and the development of atrophy alongside the response to treatment. Fluorescein angiography (FFA), indocyanine green angiography (ICG) and, increasingly, optical coherence tomography angiography (OCTA), are useful for diagnosing and monitoring neovascular disease.

With recent FDA approval of pegcetacoplan (Syfovre, Apellis Pharmaceuticals) and avacincaptad pegol (Zimura, Iveric Bio), we now have the first treatments emerging for geographic atrophy. This is likely to be followed by a range of new treatments targeting the complement-mediated inflammatory pathways in AMD. The aim of this review is to characterise the role of inflammation in non-neovascular AMD and to outline the clinical trials currently underway or completed and the therapeutic landscape of geographic atrophy.

## 2. Method

A literature search was performed to identify relevant studies for the scope of this narrative review. Databases including Pubmed, Medline, Scopus and Web of Science were searched with appropriate keywords [AMD OR Age-related macular degeneration OR dry AMD AND inflammation OR inflammatory OR complement OR immune AND clinical trials OR treatment OR therapy]. The search excluded articles not published in English. Further, the reference lists were manually reviewed. The narrative review involved a qualitative synthesis of the articles and sources that were of an adequate standard and considered relevant.

## 3. The Role of Inflammation in the Pathogenesis of Geographic Atrophy in AMD

There are widely accepted risk factors for AMD which include ageing, genetic and environmental risk factors. In the aging retina, there is building evidence of para-inflammation in the retina over time [10]. There are many genetic factors, such as, *C3*, *CFH* and *CFHR3*, that increase the risk of late-stage AMD reported by genome-wide association studies (GWAS) [11,12]. Environmental risk factors, such as smoking, diet and exercise, also result in oxidative stress on RPE cells [13]. The convergence of these risk factors is believed to increase the risk of inflammation which has been strongly associated with the pathogenesis of AMD [14]. Deposits of lipoproteinaceous material called soft drusen accumulate in the subretinal space and in the sub-RPE space. These deposits of lipoproteinaceous material have been suggested to drive RPE stress and retinal inflammation. In AMD, inflammation contributes to the damage and death of photoreceptor cells and RPE. We will describe the role of the complement pathway, interplay of microglia and neutrophils, oxidative stress and lipid metabolism in AMD pathogenesis.

### 3.1. Complement Pathway

**Figure 2:**
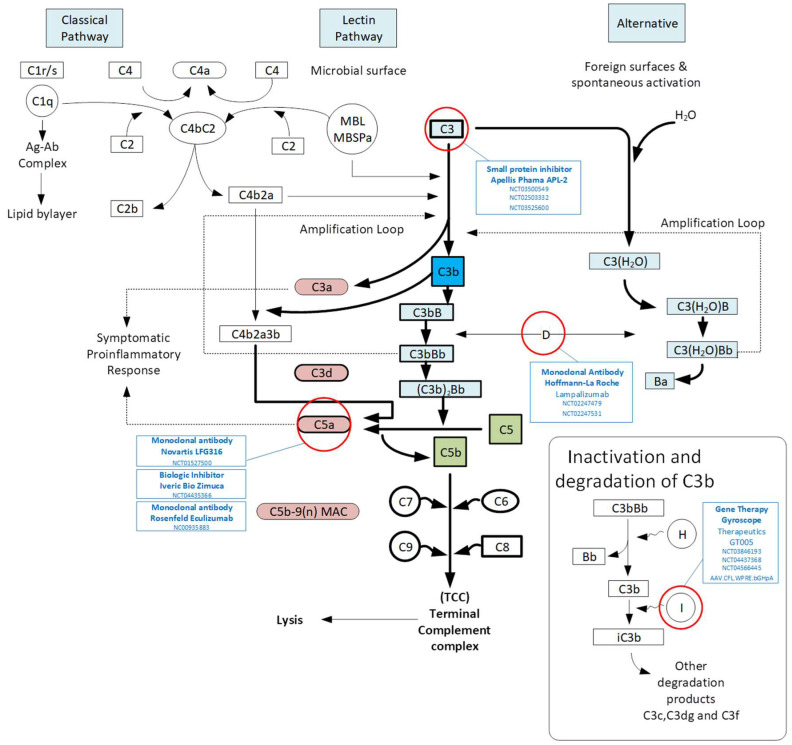
The complement pathways depicting the potential treatment targets in geographic atrophy. Dysregulation of complement factors has been associated with AMD, and complement C3 is the point of convergence of the classical and alternative pathways. Several complement pathway targets have been identified and explored in current clinical trials (circled red): C3 (a small molecule inhibitor called APL-2, pegcetacoplan, Apellis), complement factor D (a monoclonal antibody lampalizumab, Hoffmann-La Roche), complement factor I (gene therapy GT005, Gyroscope Therapeutics), CD59 (gene therapy AAVCAGsCD59, Janssen Research) and C5 (a monoclonal antibody LFG316, tesidolumab, Novartis; eculizumab, University of Miami).

There is growing genetic evidence to suggest that the complement pathway has a role in AMD. It consists of three main pathways: classical, lectin and the alternative pathways (Figure 2). All three complement pathways result in the formation of C3 convertase, with subsequent cascade resulting in a membrane attack complex (MAC) that leads to cell lysis, but each pathway differs in its initiation and regulatory components. The classical pathway is typically activated by the binding of C1 complex to antigen-bound antibodies, which leads to the activation of C2b and C4b. The lectin pathway is activated by lectin, a group of proteins that bind to carbohydrate moieties that are part of glycoproteins and glycolipids, thus serving as pattern recognition receptors to activate downstream complement cascade. Finally, the alternative complement pathway is activated at a basal level through the spontaneous hydrolysis of C3, and can be further activated by direct contact with pathogenic surfaces (e.g., microbial). It can be inactivated by complement factor H (CFH) or factor I (CFI). All complement pathways converge on the production of effector molecules, C3a and C5a, which have been shown to be present in drusen in AMD [15,16].

Complement factor H (CFH) has an important role in regulating the complement system, in particular the alternative pathway, with genetic variants associated with an increased risk of AMD [17]. Extracellular CFH protects host cells from attack by the complement pathway to prevent lysis of the host cell and chronic inflammation [18]. It does so by inhibiting C3 cleavage into C3a and C3b. Elevated C3a and C3b causes cleavage of C5 into C5a and C5b and the formation of MAC on the cellular surface. This results in lysis and chronic inflammation. Intracellular CFH acts as a cofactor for cathepsin L, cleaving C3 into C3a and C3b [18].

The concentration of complement activation products has been found to be higher in the plasma of AMD patients [19]. In addition, several components of complement factor C3 have been identified in drusen located in the sub-RPE space and choroid of AMD patients [20]. Furthermore, post-mortem studies have identified C5 in drusen and in the sub-RPE space [21]. The alternative complement pathway is continuously activated, and tissue surfaces require continuous complement inhibition to minimise cell injury. Host cells use membrane-integrated and surface-attached plasma regulators to alter the complement activation at the surface [22]. The central role for the complement pathway in AMD was illustrated by genome-wide association studies, which demonstrated that variants in several complement factors such as *CFH*, *CFB*, CFI, *C2* and *C3* increased the risk of developing AMD [23,24]. One of these single nucleotide polymorphisms (SNPs), rs1061170, in CFH results in a tyrosine-to-histidine amino acid substitution at position 402 (Y402H) [25,26]. Homozygous CC individuals have been shown to have a 5.52 fold increased risk of AMD [27]. 

The accumulation of complement proteins may drive cyclical local inflammatory responses and AMD pathogenesis. Specifically, complement factor I (CFI) is a key enzyme of the complement system which prevents positive amplification of the alternative pathway and uncontrolled accumulation of complement proteins. Certain SNPs in CFI can predispose individuals to more aggressive amplification loops increasing the risk of developing AMD [28,29]. Thus, a rare, highly penetrant missense mutation encoding Gly119Arg substitution in CFI has been associated with a high risk of AMD [30,31]. In addition, a genome-wide association study of AMD demonstrated that an increased burden of CFI variants was associated with an earlier onset and more severe AMD disease and low serum CFI levels in the presence of rare CFI variants were found to be associated with a much higher risk of developing advanced AMD [31].

### 3.2. Microglia and Neutrophils

The RPE has a role in the activation, migration and infiltration of immune cells such as microglia, monocytes and macrophages into the subretinal space [32]. Microglia have a critical role in the retina both in homeostasis and degeneration [33]. It has been demonstrated that RPE cells stimulate neutrophil activation and infiltration into the retina in a geographic atrophy mouse model with a knockout of *Cryba1* gene in RPE [34]. RPE-derived factors activate microglial cells and a pro-inflammatory (M1) degradation through Akt2 activation in microglia [34]. The microglia cells activate neutrophils as suggested by the formation of neutrophil extracellular traps with raised lipocalin-2 (LCN-2) and myeloperoxidase levels [35]. Pro-inflammatory microglia upregulate adhesion factors, including integrins beta-1 and alpha-4, on neutrophils, which assist with transmigration into the tissue through activation of CD14 on microglia and beta-1/alpha-4 on neutrophils [34]. Interestingly, targeting Akt2 in microglia with an inhibitor decreased the pro-inflammatory transition in microglial cells and neutrophil activation, indicating a possible novel therapeutic target for early AMD [34].

Microglia, the local resident immune cells in the retina, have been suggested to have both an adaptive response to eliminate toxic waste products and maladaptive response to exacerbate retinal degeneration [36]. There are two distinct microglia pools that differ in IL-34 dependency and niche [37]. In the retinal homeostasis, IL-34-dependent microglia help with neuronal function, and in degradation, localize to the RPE [37]. This transition results from the reprogramming of microglia with decreased expression of checkpoint genes and upregulation of pro-inflammatory genes. Genetic polymorphisms—for example in *CX3CR1*— have been associated with AMD [38,39].

Previous studies have demonstrated a significant increase in the number of neutrophils in the blood of neovascular AMD patients. The resolution of inflammation is associated with downregulation of the inflammatory marker CXCR2 on neutrophils [40]. Similarly, in geographic atrophy patients and the conditional *Cryba1* knockout mouse model of geographic atrophy, neutrophils infiltrate the retina and are associated with chronic inflammation [35]. It has been suggested that Akt2 signalling in RPE triggers neutrophil infiltration in AMD patients and in mouse models with retinal degeneration. Akt2 inhibition in a mouse model decreased neutrophil infiltration and subdued RPE changes, supporting the role of Akt2 as a potential therapeutic target [35].

### 3.3. Oxidative Stress Mediated Inflammatory Responses

Oxidative stress, resulting from the imbalance between the production of reactive oxygen species (ROS) and the neutralisation of their harmful effects, is linked to the inflammatory pathways in AMD [41]. Oxidative stress results in a breakdown of the outer blood–retinal barrier allowing immune factors/cells to infiltrate. Chronic oxidative stress causes impaired physiological function and autophagy in RPE [42]. Oxidative processes lead to the removal of electrons and production of reactive oxygen species—for instance, free radicals and hydrogen peroxide. Oxidative stress and inflammation are strongly related as inflammatory cells can produce reactive oxygen species, and oxidative stress results in inflammation through NF-kB mediated gene expression [43].

A proteomic analysis of drusen from AMD donor eyes and controls identified multiple proteins altered by oxidation, including vitronectin, carboxymethyl lysine and tissue metalloproteinase inhibitor 3 [44]. It was also observed that there was an increased level of oxidation product-related receptor in advanced AMD. Another study showed 60% of proteins with raised levels were implicated in immune response which included complement factors C5 and alpha crystallin A [45].

Oxidative stress predisposes RPE cells to complement-mediated injury [46]. The RPE is susceptible to oxidative stress, which leads to intracellular accumulation of lipofuscin in the RPE and extracellular drusen between Bruch’s membrane and RPE cells [47]. There are three theories explaining the association between oxidative stress and complement pathway activation: (1) oxidative stress regulates *CFH* and *CFB* expression, (2) the H402Y variant cannot generate anti-inflammatory iC3b components on malondialdehyde-loaded surfaces, and (3) phagocytosis of oxidized photoreceptor outer segment (POS) material disrupts the synthesis of *CFH*. A preclinical in vivo model has linked oxidative stress with complement activation in retinal degeneration [48]. A significant risk factor for AMD is cigarette smoking, and this causes increased oxidative stress through several mechanisms. It causes both direct damage via free radicals and induces an inflammatory response [49,50].

### 3.4. Lipid Metabolism and Inflammation in AMD

Several lines of evidence implicate altered lipid metabolism in the pathogenesis of AMD. Firstly, lipids are an abundant component of drusen, the hallmark feature of AMD, and account for 40% of drusen volume [51]. Drusen, and other drusenoid deposits such as reticular pseudodrusen, are hypothesised to develop as a result of abnormal lipid cycling between the photoreceptors, RPE and choriocapillaris [52]. An age-related accumulation of lipid and lipoprotein, the so-called ‘oil spill’ [53] in Bruch’s membrane, is proposed to impair normal lipid exchange, further leading to a build-up of lipoproteinaceous debris that can act as oxidisation targets as well as foci for complement activation and inflammation. 

Secondly, multiple GWAS have demonstrated that genetic variants within or near genes involved in lipid metabolism pathways, including *APOE*, *LIPC*, *ABCA1* and *CETP,* are associated with AMD risk [23]. In particular, *APOE* is a lipid transport protein found in drusen that is expressed locally by the RPE, as well as systemically by the liver. It known to activate the complement system, promote subretinal inflammation via mononuclear phagocyte accumulation and interact with amyloid-beta, another important component of drusen [14]. Key murine models of AMD include transgenic mice with null or altered function in genes important for lipid transport—for example, *APOE*, *APOB100*, *ABCA1*, *CD36* and low-density lipoprotein receptor [54,55].

Thirdly, obesity [56] and a diet high in saturated fat [57] are associated with AMD, and while an altered serum lipid profile has been linked to AMD, the relationship is inconsistent [58,59]. Converse to traditional attitudes towards high-density lipoprotein (HDL) as a ‘good cholesterol’, epidemiological studies into the association between HDL and AMD in fact demonstrated an odds ratio increase of 1.21 per 1-mmol/L increase in HDL, and higher HDL levels led to greater odds of larger drusen size [60,61]. Notably, a high fat diet is a common environmental variable used to precipitate the disease phenotypes (Bruch’s membrane thickening and subretinal deposits) found in the aforementioned mouse models. Meanwhile, closer adherence to a Mediterranean diet was correlated with a lower risk to advanced AMD [61]. Given the strong involvement of cholesterol and lipid metabolism in AMD pathology, statins have been studied for the treatment of AMD not only for their cholesterol-lowering effect but also anti-oxidant and anti-inflammatory properties [62]. Currently, evidence that statins could prevent or delay AMD onset or progression are conflicting, possibly relating to limiting high doses required to achieve any therapeutic benefit in the retina [63]. They may constitute a promising therapeutic modality for future development.

## 4. The Current Landscape of Dry AMD Therapeutic Strategies: Slowing down the Progression

Over the last few decades, there have been major efforts to develop new therapies for geographic atrophy which slow down the rate of disease progression by targeting the retinal immune system. Advances in strategies to treat AMD include small molecule inhibitors, monoclonal antibodies and gene therapy that have been developed to target the complement, visual cycle, neuro-protection, photo-biomodulation and oxidative stress pathways (Table 1). There are multiple clinical trials at different phases and stages outlined below with the aim to slow down or halt disease progression or restore vision.

### 4.1. Complement Inhibition

There have been multiple clinical trials targeting the complement pathway because it has a fundamental role in the pathogenesis of dry AMD (Figure 2). Complement factor 3 was targeted since it has a central role in the activation of the complement cascade [64]. In addition, high levels of C3 have been found in drusen, which is a hallmark of disease. Pegcetacoplan (APL-2), a pegylated derivative of cyclic tripeptide compstatin, binds and blocks its cleavage into C3a and C3b [65]. This decreases inflammation associated with anaphylatoxin C3a formation and antigen uptake through antigen-presenting cells and breakdown of C5. Multi-centre randomised controlled trials of pegcetacoplan were performed by Apellis. OAKS (NCT03525613) and DERBY (NCT03525600) have completed phase III studies with a reduction in geographic atrophy lesion growth. Pegcetacoplan has been FDA approved.

Targeting complement factor 5 has an advantage over blocking C3 since it can contribute to host defence while simultaneously inhibiting inflammasome activation and MAC formation. At the terminal complement activity, complement factor 5 inhibitors have been developed. Tesidolumab (LFG316), a monoclonal antibody, LFG316 was developed to inhibit C5 and prevent cleavage into C5a and C5b. A phase II multicentre randomised control trial of LFG316 was performed in geographic atrophy (NCT01527500) and showed no difference at 12 months. Eculizumab similarly inhibited C5 cleavage, and the COMPLETE study randomised patients with a high-dose compared to placebo and demonstrated no difference in geographic atrophy growth with follow up at 6 and 12 months [66]. Further targeting C5, avacincaptad pegol (Zimura, Iveric Bio; NCT04435366) worked to prevent MAC and inflammasome formation [67]. The randomised control trial GATHER1 assessed the safety and efficacy with demonstrated geographic atrophy growth reduction. Avacincaptad pegol has been FDA approved. 

Complement factor I has been supplemented through local ocular AAV-mediated gene therapy with the aim to suppress the alternative complement pathway within the eye [68]. This gene therapy (GT005) led by Gyroscope and now Novartis is currently in a Phase II trial (NCT03846193) for geographic atrophy and has received fast track designation from the FDA. It has been developed as a once-only treatment with the aim of decreasing inflammation and preventing further vision loss by slowing the progression of geographic atrophy.

The GOLDEN study is targeting Complement factor B to investigate a novel approach of targeting a specific gene in geographic atrophy in AMD. The inhibitor IONIS-FB-Lrx was used to target the RNA antisense of complement factor B in the alternative pathway (NCT03815825) [69]. The formation of MAC can be inhibited by complement factor CD59 to prevent the addition of C9 to the complex. Gene therapy HMR59 (AAVCAGsCD59) is being developed to induce retinal and RPE cells to express a soluble version of CD59. It uses the AAV2 vector delivery system with the safety and efficacy assessed in geographic atrophy (NCT03585556).

### 4.2. Neuroprotection

Neuroprotective agents have been explored in preclinical studies to halt the progression of geographic atrophy in AMD. This includes ciliary neurotrophic factor (CNTF) using encapsulated cells implanted intravitreally to treat retinal degeneration. A multicentre, dose-ranging phase II study reported results of geographic atrophy patients with an implant removed after 6 months which showed reduced progression of vision loss [70,71]. Another agent with neuroprotective properties is Brimonidine, which primarily acts as an alpha2 adrenergic receptor agonists to lower the intraocular pressure. In a phase IIb study (BEACON study), the geographic atrophy area decreased in the treatment group. However, it was terminated early because of slow geographic atrophy progression in the control group.

### 4.3. Visual Cycle Modulation

It has been suggested that targeting the visual cycle, in which vitamin A has a central role, may be a further therapeutic approach [72]. In AMD, vitamin A monomers dimerize to form bisretinoid, which accumulates as lipofuscin in the outer retina and RPE [73]. Various visual cycle modulators aim to decrease the accumulation of toxic fluorophores in RPE cells. Emixustat has been used to inhibit the production lipofuscin and A2E from the visual cycle [74,75]. Its efficacy was measured by ERG rod sensitivity following photobleaching [75]. Following 2 weeks of administration, there was a dose-dependent suppression of rod ERG, although no significant change was measured in cone ERG. It was reported that 93% of patients subjected to Emixustat had an ocular adverse event which was most commonly visual disturbances, and 57% had systemic adverse events [74]. A deuterated form of vitamin A (ALK-001) has been developed to dimerize at a delayed rate while preserving function [76]. A phase III trial (NCT03845582) aims to assess the efficacy based on the rate of geographic atrophy progression in AMD.

### 4.4. Glyco-Immune Modulation

The accumulation of drusen activates pro-inflammatory activity which has involved glycans that have a role in immune regulatory activity. Aviceda have developed a means to target mimic self-associated molecular patterns (SAMPS) glycans that regulate the immune system. Two therapeutic strategies involve inhibiting the inflammatory cells while in a resting state and in complement activation. A nanoparticle called AVD-104 was developed and is being evaluated to treat geographic atrophy [77]. Recently, it was announced that that the US FDA have cleared AVD-104 for phase II clinical trial (called SIGLEC) for geographic atrophy.

## 5. Future Possible Therapeutic Strategies to Treat Geographic Atrophy: Vision Restoration

**Figure 3:**
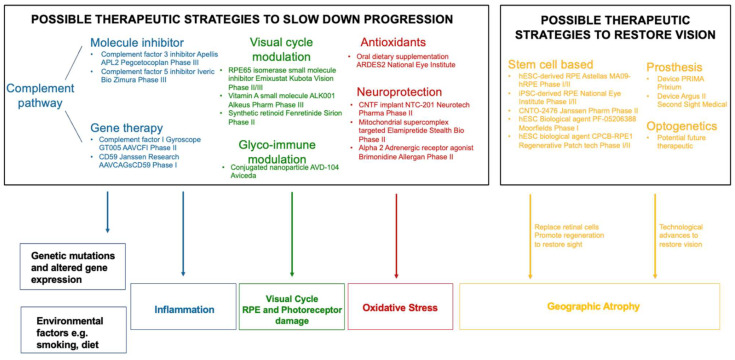
Summary of the potential therapeutic strategies to slow down progression and restore vision in geographic atrophy.

While inflammation has a significant role in AMD pathogenesis, in many patients there has already been extensive retinal damage which means that alternative approaches may be needed in more severe cases. Most clinical trials for GA treatment aim to slow down the rate of disease progression. In addition, some advances have been made to develop treatments to restore visual function in the retina, after photoreceptors and RPE have become irreversibly atrophic. (Figure 3). Such advances through stem cells, prosthetic devices and optogenetics will be further discussed.

### 5.1. Cell Therapy

Stem cell therapy is being developed as a means to replace dysfunctional retinal cells, promote regeneration and remodelling and improve visual function. There are two approaches in stem cell therapy: paracrine and cell-replacement therapy [78]. Paracrine therapy uses diffusible factors that promote the function and viability of surviving photoreceptors. It includes umbilical tissue cells and bone marrow stem cells that originate from the mesenchymal stem cell line. Meanwhile, the stem cell replacement strategy aims to replace RPE. The RPE cells derived from human embryonic stem cells (hESCs) and human induced pluripotent stem cells (iPSCs) are administered directly into the subretinal space adjacent to the area of geographic atrophy. The transplanted RPE cells thus can give nutritional and metabolic support to the nearby photoreceptors to maintain their photoreceptive function.

Subretinal injection of hESC-derived RPE (50,000–200,000 cells into the eye) has been trialled for geographic atrophy [79]. The transplantation of hESC-derived RPE cells (MA09-hRPE) was considered safe and tolerable [80]. There were no adverse events, including proliferation, serious ocular or systemic side effects or rejection of the transplanted cells. In the study of 18 patients, the best-corrected visual acuity improved in 10 eyes, was constant in 7 eyes, and decreased by more than 10 letters in 1 eye, while the untreated fellow eyes showed no improvements. Quality of life improved for peripheral and general vision by 16 to 25 points over 3 to 12 months after transplantation in patients with geographic atrophy [79].

A polarised monolayer of hESC-derived cells (CPCB) was administered on a parylene membrane into patients with geographic atrophy (phase I/II study; NCT02590692). Preliminary results suggest successful implantation into four patients and visual acuity improvement of 17 ETDRS letters in an individual patient.

Finally, human umbilical cord tissue derived cell compound called CNTO-2476 or Paluocorcel has been demonstrated to decrease functional deterioration in a rat model of geographic atrophy. In a phase I/II study, using an iTrack Model microcatheter, CNTO 2476 was administered subretinally into patients with geographic atrophy. While there was a high rate of adverse events associated with the delivery system, there was an improvement in 34.5% of patients of more than 10 letters at 1 year follow up.

### 5.2. Prosthesis

In addition to cell therapy, a prosthetic system is currently under trial in geographic atrophy called is PRIMA, a photovoltaic subretinal wireless bionic vision device [81,82,83]. It comprises a camera to capture visual information, a pocket processer attached to glasses and a transmitter to a small projector that uses pulsed infrared light onto the subretinal implant. The architecture of this device is aimed at reducing size and the complexity. The photovoltaic cells convert visual information into electrical signals to stimulate the retina. A clinical trial with five participants with geographic atrophy has reported its findings thus far [83]. The eligibility criteria included participants age over 60 years, GA due to AMD in both eyes, study eye has best corrected visual acuity of logMAR 1.2 or worse and an atrophic patch of at least the implant size (NCT04678854). After follow up, all five could perceive white-yellow prosthetic visual patterns where previously there were scotomas [83]. Further research is needed with longer follow up.

### 5.3. Optogenetics

Optogenetics is another potential future treatment for advanced AMD. It uses optical and genetic engineering strategies to introduce light-sensitive proteins into inner retinal cells that are normally insensitive to light [84]. These surviving cells could then provide a pathway to bypass the degenerated RPE and photoreceptors and restore vision [85]. Despite the structural and functional remodelling in advanced geographic atrophy, the remaining inner retinal neurons may be receptive to optogenetic opsin expression and light-evoked responses. Optogenetic therapies in development focus on restoring vision in advanced retinal degeneration, via gene agnostic approaches [86]. Currently, the only reported trial is the PIONEER study by Gensight Biologics which has shown a good safety profile with 2.5 years follow up and preliminary partial functional recovery in two patients [87]. Although current clinical trials have recruited patients with advanced pan-retinal degeneration, there is no reason why this therapy could not be applied to geographic atrophy.

Optogenetics takes advantage of the surviving neural architecture that is receptive to light, and while geographic atrophy and inherited retinal disease both result in atrophy and, ultimately, vision loss, the mechanisms and progression of these two diseases are somewhat different. In classic rod-cone dystrophy, the commonest type of inherited retinal disease, there is typically loss of rod followed by loss of cone photoreceptors, typically starting in the periphery and ultimately affecting the central macular area. The inner retinal cells survive for a long time following this loss, and the bipolar cell becomes the highest order neuron which can be altered by optogenetics to become the light-detecting cell. In geographic atrophy, the outer retina can in addition be directly altered by drusen deposits and inflammation, resulting in cell death of primarily cone photoreceptors which affects colour and central vision, as well as the death of the RPE that support photoreceptors. In geographic atrophy, there is some evidence that the inner retina becomes thinner in earlier stages of the degeneration compared to classic rod-cone dystrophies [86]. Thus, the bipolar cells may be affected sooner in geographic atrophy, and the retinal ganglion cells in the innermost layer of the retina could be a more suitable target in geographic atrophy.

## 6. Conclusions

An understanding of the role of inflammation will provide insight into the pathways that can be therapeutically targeted to slow or prevent progression of non-neovascular AMD (Figure 3). The development of future AMD treatment depends on an improved and growing understanding of the disease pathogenesis, which can guide personalised patient care and may involve a combination of approaches.

The ideal therapy for geographic atrophy needs to be safe and effective with cost benefits for patients and health care providers. Early phase clinical trials have demonstrated promising results with anticipated potential for future therapies. We now have the FDA approved-therapeutics, pegcetacoplan (Syfovre) and avacincaptad pegol (Iveric Bio), for geographic atrophy. However, it remains to be seen how much long-term benefit this treatment will provide to the patients considering the frequency of injections if given monthly with little or no apparent benefit for years to follow after treatment. A one-off gene therapy treatment would hence be a very attractive option.

The significant and increasing burden of disease of AMD highlights the need for the rapid development of additional therapeutics aimed at slowing down the degeneration and restoring sight. It is important to recognise that patients with neovascular AMD can also develop geographic atrophy. Therefore, even when neovascular disease is successfully treated, vision loss may still occur due to geographic atrophy. Further investigation is needed to determine the feasibility of restorative technologies to be applied to geographic atrophy, once the disease is too advanced for rescue therapies. Specifically, the viability of the administration of stem cells, retinal prostheses and optogenetics to reverse vision loss in geographic atrophy requires further investigation. After demonstrating the efficacy of optogenetics in RP, there is potential that it could also have a role in the treatment of geographic atrophy.

## Figures and Tables

**Table 1:** Approaches for the treatment of geographic atrophy (GA). Approaches include targeting complement protein, glyco-immune therapeutics, visual cycle modulators, neuroprotection, stem cells, prosthesis.

Approach	Institute/Company	Target/Therapeutic	Phase, NCT, Status, Year and Studies	Outcome/Comment
Complement	Apellis Pharma	C3; Small molecule inhibitor APL2 Pegcetacoplan	Phase III, NCT03525600, Active, started 2018, DERBY, OAKS	FDA approved. GA growth rate significantly decreased
	Iveric Bio	C5; Biologic inhibitor Zimura	Phase III, NCT04435366, Active, started 2020, GATHER2	FDA approved. GA growth rate significantly decreased
	Hoffmann-La Roche	CFD; Monoclonal Ab Lampalizumab	Phase III, NCT02247479, Terminated 2019, CHROMA, SPECTRI	Failed to slow down GA growth
	Alexion	CFD; Small molecule inhibitor Danicopan	Phase II, NCT05019521, Recruiting 2021	Not available yet
	Gyroscope Therapeutics	CFI; Gene therapy GT005	Phase II, NCT03846193, Active, started 2018, EXPLORE, HORIZON	EXPLORE includes rare variants of CFI, HORIZON with GA
	Ionis Pharmaceuticals	CFB; Antisense inhibitor IONIS-FB-LRx	Phase II, NCT03815825, Active, started 2019, GOLDEN	Not available yet
	National Eye Institute	Rapamycin	Phase II, NCT00766649, Completed 2012, SIRGA	No anatomical or functional benefit
	Allegro Ophthalmics	Risuteganib	Phase II, NCT03626636, Completed 2019	Significant BCVA improvement in dry AMD over 32 weeks
	University of Miami	C5; Monoclonal Ab Eculizumab	Phase II, NCT00935883, Completed 2012, COMPLETE	Failed to slow GA progression
	Janssen Research	CD59; Gene therapy AAVCAGsCD59	Phase I, NCT03144999, Completed 2019	Decreased GA growth rate at 6 months
Visual cycle modulator	Alkeus Pharm	Vitamin A; Small molecule compound ALK-001	Phase III, NCT03845582, Active, started 2019, SAGA	Not available yet
	Kubota Vision	RPE65 isomerase; Small molecule inhibitor ACU4429 Emixustat	Phase IIb/III, NCT01802866, Completed 2016, SEATTLE	Did not reduce GA growth
	Sirion Therapeutics	Synthetic retinoid compound; Fenretinide	Phase II, NCT00429936, Completed 2010	Not available yet
Antioxidant	National Eye Institute	Oral dietary supplementation	Phase III, NCT00345176, Completed 2012, ARDES2	High levels of antioxidants and zinc can decrease risk of advanced AMD by 25%
Neuro-protection	Neurotech Pharmaceuticals	CNTF implant NTC-201	Phase II, NCT00447954, Completed 2009	Favourable pharmacokinetic profile without systemic exposure
	Stealth Bio Therapeutics	Mitochondrial supercomplex; Small peptide Elamipretide	Phase II, NCT03891875, Completed 2022, ReCLAIM-2	Categorical >2 line improvement in low luminance visual acuity in GA
	ONL Therapeutics	ONL1204; Apoptosis inhibitor	Phase I, NCT04744662, Active, started 2021	Not available yet
	Allergan	Alpha2-adrenergic receptor agonist, Brimonidine	Phase IIb, NCT02087085, Terminated 2018	Terminated due to lower than expected GA progression in the control group.
Anti-Inflammatory	Paul Yates	30S ribosomal subunit, antibiotic Doxycycline	Phase II/III, NCT01782989, Completed 2022 TOGA	Not available yet
	National Eye Institute	Oral minocycline	Phase II, NCT02564978, Completed 2022	No significant difference in GA enlargement rate between run-in and treatment phase
	Genentech	GM-CSF; Monoclonal Ab Galegenimab FHTR2163	Phase II, NCT03972709, Terminated 2022, GALLEGO	Terminated because the benefit to risk did not support further treatment
	Ocugen	OCU410 Orphan nuclear receptor; Modified gene therapy	Not available	Not available
Photo-biomodulation	LumiThera	Light Delivery system LT-300	NA, NCT04522999, Completed 2021, LIGHTSITE III, ELECTRO-LIGHT	Positive multi-luminance ERG and visual acuity improvement (58.2% of treated eyes improved >5 letters in 13 months)
Glyco-immune	Aviceda Therapeutics	SIGLEC; Conjugated nanoparticle, AVD-104	FDA approved AVD-104 to initiate Phase II; SIGLEC	Not available yet
Cell therapy	Astellas Pharma	hESC-derived RPE (MA09-hRPE)	Phase I/II, NCT01344993, Completed 2021	Medium-term to long-term safety, graft survival and possible biologic activity
	National Eye Institute	iPSC- derived RPE	Phase I/II, NCT04339764, Recruiting	Not available yet
	Janssen Pharm	IL-12 and IL-23, Monoclonal Ab, CNTO-2476	Phase II, NCT02659098, Completed 2020, PRELUDE	Mild ocular AE but no demonstrated efficacy in GA area growth or VA
	MD stem cells	Bone marrow derived stem cells (BMSC)	NA, NCT03011541, Recruiting, SCOTS2	Not available yet
	Tenpoint Therapeutics	hESC-derived RPE patch graft PF-05206388	Phase I, NCT01691261, Recruiting	Not available yet
	Regenerative Patch Tech	hESC-derived RPE patch graft CPCB-RPE1	Phase I/II, NCT02590692, Active	Not available yet
Prosthesis	Second Sight Medical	Device Argus II System	NA, NCT02227498, Completed 2020, ArgusII	No significant change in visual acuity and 4 SAE
	Pixium Vision	Device PRIMA	NA, NCT03392324 PRIAM-FS-US	3 out of 5 patients had BCVA improvement from 20/460 to 20/550 in 12 months
